# Chromium (VI) Ion Adsorption Features of Chitosan Film and Its Chitosan/Zeolite Conjugate 13X Film 

**DOI:** 10.3390/molecules16053569

**Published:** 2011-04-28

**Authors:** Anabelle C. L. Batista, Emílio R. Villanueva, Rosa Valéria S. Amorim, Maria Teresa Tavares, Galba M. Campos-Takaki

**Affiliations:** 1 Rede Nordeste de Biotecnologia (RENORBIO), Universidade Estadual do Ceará, Av. Paranjana, 1.700, Campus do Itaperi, 60740-000 Fortaleza, CE, Brazil; Email: ellecamarotti@yahoo.com.br; 2 Núcleo de Pesquisas em Ciências Ambientais (NPCIAMB), Universidade Católica de Pernambuco, Boa Vista 50 050-590 Recife, PE, Brazil; 3 Departamento de Engenharia Química, Universidade de Vigo, Spain; Email: emiliorv@uvigo.es; 4 Departamento de Histologia e Morfologia, Universidade Federal de Pernambuco, Av. Prof. Moraes Rego, 1235, Cidade Universitária, 50670-901, Recife, PE, Brazil; Email: rosa.amorim@ufpe.br; 5 Instituto de Biotecnologia e Bioengenharia (IBB), Universidade do Minho, Campus de Gualtar, 4710-057 Braga, Portugal; Email: ttavares@deb.uminho.pt

**Keywords:** chitosan, zeolite, chromium, adsorption

## Abstract

This research evaluated the importance of the adsorption properties of chitosan a chitosan/zeolite conjugate film for the removal of Cr(VI) ions from solutions in the 5–260 mg/L concentration range, when the pH was adjusted to 4.0 and 6.0. The uptake capacities of the films formed by chitosan and by the chitosan/zeolite conjugate were calculated by mass balance. The equilibrium isotherms were fitted to the Langmuir, Freundlich and Redlich-Peterson models. The chitosan film seems to be a good sorbent for Cr(VI) at pH 4, but its physical instability suggests the need for a more resilient support. Due to this fact zeolite was added to the chitosan matrix in solution and a chitosan/zeolite (CS/Zeo) film was thus formed. The solubility of the film and the characterization of the different matrices by FTIR, TGA and X-Ray showed that a cross-linked structure was formed between the chitosan and zeolite and the solubility of the film increased. In this study, the low manufacturing cost of the CS/Zeo matrix, the good uptake of Cr(VI) at acidic pH (17.28 mg/g) and the non desorption of Cr(VI) from the film in water suggests this combination should be tested in industrial environment.

## 1. Introduction

Chitin (CH) has been widely used for cosmetics, feed additives and so on due to the fact it is a harmless and inexpensive material, and the good adsorption performance of chitin has been shown. Nowadays, several technologies using microorganisms, chitosan (CS) or zeolites (Zeo) have been tested as ways of removing heavy metals from the environment [1,2,3,4,5,6]. During the adsorption process by CS, the nitrogen from the amino group of CS acts as an electron donor is thought to be mainly responsible for the adsorption of metal ions, although metals can also bind with the free hydroxyl groups in the CS molecule [7]. Zeolite is a naturally occurring mineral group consisting of over 50 different minerals. The 13X zeolite was chosen to crosslink with CS because it shows excellent stability in acid solutions, supports high temperatures and it has been already tested as a non Cr(VI) adsorbent [8]. This combination of substances (CS/Zeo) to form a film has been successfully used in pervaporation experiments with organic-water systems [9,10,11].

In this study, the heavy metal adsorption efficiency of CS was combined with the excellent physico-chemical properties of the zeolite to improve film performance. The chemical changes resulting from these combinations were investigated and the adsorption/desorption experiments were evaluated using Cr(VI), a metal commonly found in industrial wastewaters. The adsorption isotherms of Cr(VI) ions on CS and CS cross linked with Zeo films were studied for purposes of comparison. 

## 2. Results and Discussion

### 2.1. Film characterization

The preliminary results obtained with CS film (Table 1) showed that its solubility in water increases when the chitosan concentration on the film increases. This is justified by the high hydrophilicity of chitosan, which is dependent on the presence of –NH_2_ and –OH groups. The increased amount of intermolecular bonds between free reactive groups of internal residues of chitosan is also suggested as a facilitator of increased resistance to solubilization and the resistance to degradation of the film [12,13]. The CS/Zeo films demonstrated good physical stability in metal solutions, being more soluble at pH 4.0 than pH 6.0, depending on the metal concentration in solution (Table 1). The decreased solubility of CS 0.5% and CS/Zeo films in solutions ranging from 5 mg/L to 260 mg/L of Cr(VI) concentration at pH 4.0 occurred presumably due to the formation of a large amount of electrostatic bonds between the reactive NH_2_ group of CS and the major HCrO_4_^−^ form of Cr(VI) at low pH, which increased the resistance to solubility. The increased solubility of the films in solutions from 5 mg/L to 260 mg/L of Cr(VI) concentration at pH 6.0 is potentially due to the lesser formation of electrostatic bonds between the reactive groups of the CS and Cr(VI), that favors the instability of the films [10,11,12]. Films with solubility higher than 35% in water and Cr(VI) solution demonstrated instability of their integrity during the assays. Enhanced resistance of chitosan to acids and chemicals was suggested previously [11,14,15].

**Table 1:** Effect of chitosan concentration, presence and metal concentration and pH onto the chitosan (CS) and chitosan/zeolite (CS/Zeo 1:10 w/w) film solubility.

Film (w/v)	Solution	Solubility	Film (w/v)	Solution	Solubility
CS 0.5%	H_2_O pH 4.0	27.71%	CS 0.5%	Cr(VI) 5 mg/L pH 4.0	23.40%
CS 1.5%	H_2_O pH 4.0	30.82%	CS 0.5%	Cr(VI) 260 mg/L pH 4.0	2.60%
CS 2.5%	H_2_O pH 4.0	33.31%	CS/Zeo	Cr(VI) 5 mg/L pH 4.0	35.96%
CS 0.5%	H_2_O pH 6.0	18.70%	CS/Zeo	Cr(VI) 260 mg/L pH 4.0	25.54%
CS 1.5%	H_2_O pH 6.0	25.93%	CS 0.5%	Cr(VI) 5 mg/L pH 6.0	12.31%
CS 2.5%	H_2_O pH 6.0	29.29%	CS 0.5%	Cr(VI) 260 mg/L pH 6.0	30.51%
CS/Zeo	H_2_O pH 4.0	18.80%	CS/Zeo	Cr(VI) 5 mg/L pH 6.0	35.81%
CS/Zeo	H_2_O pH 6.0	16.40%	CS/Zeo	Cr(VI) 260 mg/L pH 6.0	40.92%

**Figure 1:**
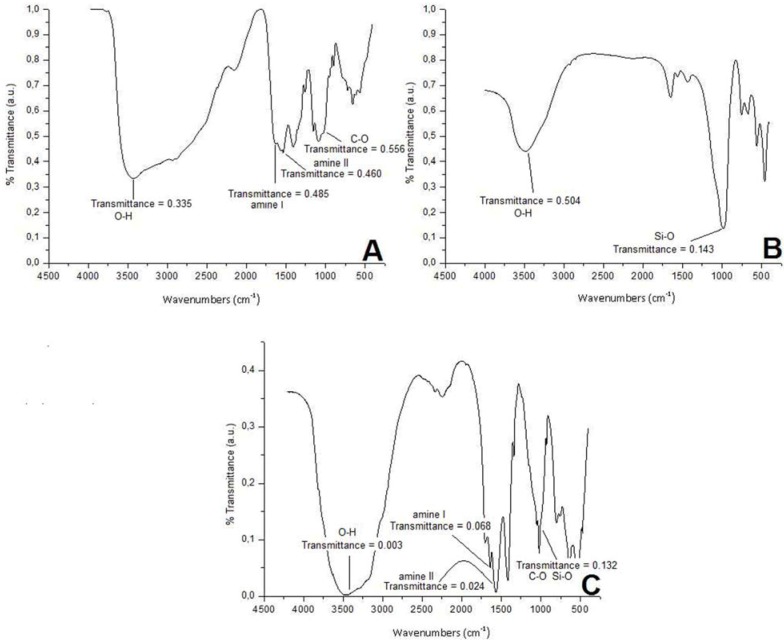
FTIR spectra of chitosan film (**A**), zeolite powder (**B**) and chitosan/zeolite film (1:10 w/w) (**C**).

In Figure 1 is possible to observe in chitosan film the bands of amine I (~1,650 cm^−1^), amine II (~1,590 cm^−1^) and C-O stretching vibrations (~1,200–1,000 cm^−1^), all characteristic of CS (Figure 1A) and the bands of Si-O and C-O stretching vibration (~1,200–1,000 cm^−1^) characteristics of Zeo on zeolite powder (Figure 1B). The O-H stretching vibration (~3,440 cm^−1^) appeared in all samples tested with different intensities according to the concentrations of reactive OH- present in the molecules. The decreased of the intensity of the characteristic peaks of CS film and Zeo powder on the CS/Zeo film is suggested by the formation of intermolecular cross-links between CS and Zeo during the formation of the film. This decrease was observed previously by Wang *et al*. [11] in different kinds of zeolite and by Kittur *et al*. [16], who described that this decrease is dependent on the zeolite content in the membrane. The weakening of C-O stretching vibration to CS/Zeo film when compared with CS film is suggested as the result of hydrogen bonds between chitosan and zeolite [11]. The absence of transmittance difference between the C-O stretching vibration on CS/Zeo film and Zeo powder suggest the incorporation of zeolite crystals in the matrice of chitosan film, which is confirmed by X-ray analysis. Intermolecular hydrogen bonds between CS and other compounds to form films were reported earlier to favor the formation of pores in the structure [17].

**Figure 2:**
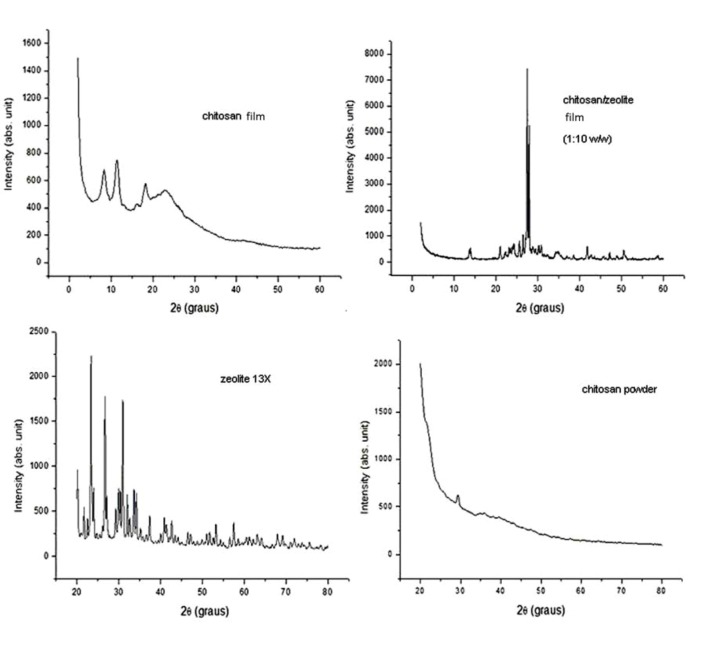
X-ray patterns of chitosan film 0.5%, chitosan/zeolite film (1:10 w/w), zeolite 13X and chitosan powder.

The X-ray diffraction studies confirmed the semi-crystallinity of CS and the crystallinity of zeolite. In this study, the CS film shows mainly the form I, where the amine groups may be involved in the stability of the semi-crystalline molecule and in the control of the accessibility to adsorption sites, as confirmed by the peak at 11.2° present in CS film 0.5% (w/v) (Figure 2). The introduction of Zeo particles into CS matrices increased the crystallinity of the film and the thermal stability, although it decreases the flexibility. The CS/Zeo film showed some characteristic peaks of both components and the differences may be justified by the incorporation of zeolite crystals in the chitosan film matrices, which may affect the adsorption process of the metal by the CS/Zeo film, as confirmed by adsorption experiments [10,11,14,16].

**Figure 3:**
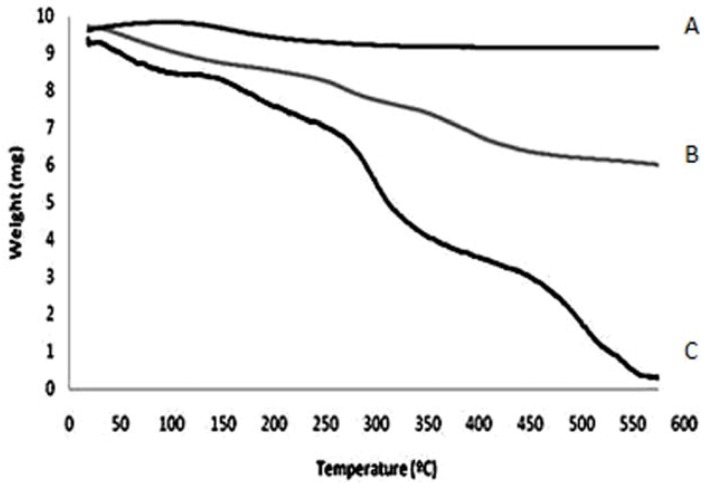
TGA results of pure zeolite powder (**A**), chitosan/zeolite film (**B**) and chitosan film (**C**).

The TGA curves show two endothermic peaks for the CS film and only one endothermic peak for the Zeo powder, as shown in Figure 3. The first peak corresponds to loss of water in all substances tested and the second peak corresponds to the degradation of the sample. This excessive loss of water of CS film in the range of 100–110 °C may limit its applications in different water systems. The higher thermal stability of the CS/Zeo film, evidenced by a gentle peak in the 110–350 °C region, suggests a low decomposition of the main chain of CS and a strong interaction between the CS and the Zeo used in these studies, as shown in Figure 3 [11,13,18,19].

### 2.2. Adsorption/desorption Experiments

The first stage of experiments aimed to analyze the adsorption of Cr(VI) at pH 6.0 by CS films prepared in three different concentrations (0.5–2.5% w/v), to select the best formulation for the association of chitosan with zeolite. The first experiments showed the physical stability of the CS film at 2.5% (w/v) in all solutions of Cr(VI). However, higher removal efficiency has been observed when 0.5% CS film was used. In order to maximize the efficiency of metal removal in solution using a batch systems and to reduce the cost of the adsorption operation we selected 0.5% (w/v) CS film to continue the experiments and for cross linking with the Zeo to form CS/Zeo 1:10 (w/w) films. The adsorption experiments with Zeo pellets demonstrated the non adsorption of Cr(VI) at different pHs or metal concentrations. The literature confirmed that there no adsorption of Cr(VI) by zeolite with unaltered surfaces, although the tailoring of zeolite surface is possible and could enable the adsorption of Cr(VI) [3,20]. 

**Figure 4:**
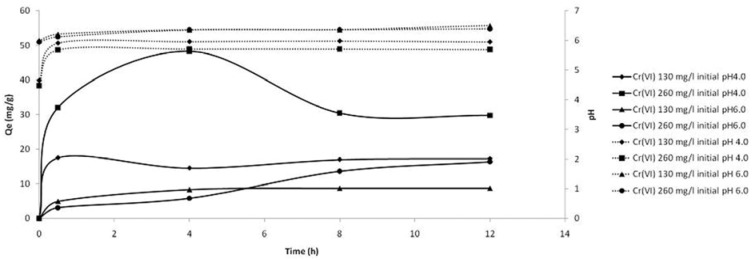
Adsorption kinetic curves for Cr(VI) on chitosan/zeolite film (full line) and evaluation of pH during assays (dotted line).

At pH 4.0 the adsorption capacity of CS film was not favored because under these pH conditions, the CS film was completely dissolved in the first thirty minutes of the experiment at concentrations of Cr(VI) lower than 130 mg/L. The incorporation of Zeo in the inner structure of the chitosan matrix, as confirmed by FTIR and X-ray, stabilized the film at concentrations that ranged from 5–260 mg/L at the initial pH values of 4.0 and 6.0. This effort to stabilize the CS film through association with other substances for use in metal adsorption was also described by other authors [10,14,15,18,19,21]. Baroni *et al*. [18] argued that quantitative analyses of Cr(VI) may present errors that are significant in batch experiments with concentrations lower than 250 mg/L using cross-linked films of chitosan. However, the CS/Zeo films used in this research study were able to stabilize the adsorption kinetics in concentrations of Cr(VI) from 130 mg/L with an adsorption uptake of 17.28 mg/g at pH 4.0 and 8.78 mg/g at pH 6.0 until equilibrium in 12 h (Figure 4). The influence of pH on Cr(VI) adsorption was described previously suggesting that the protonated form of chitosan at pH 4.0 can facilitate the chelation or electrostatic interaction with Cr(VI) in solution [18,19].

In Figure 4, it is observed that the adsorption equilibrium is rapidly reached and that it is related to the increase in pH from 4.0 to ~6.5 during the assays, which may have interfered with the protonation of the chitosan NH_2_ sites. The increase in pH is suggested by the release of Na+ from CS/Zeo film into the solution. At pH 4.0 the majority of the CS presents the protonated form NH_3_^+^ which favors the formation of electrostatic bonds in a larger amount than in systems where the pH is closer to a neutral value [22,23]. This increase of electrostatic bonds with a large amount of Cr(VI) decreased the solubility at acid pHs. At pH 6.0 the CS presents both reactive forms –NH_3_^+^/–NH_2_ and Cr(VI) the forms HCrO_4_^−^ (∼75%) and CrO_4_^2−^ (∼25%) at 25 °C, suggesting that the phenomenon of adsorption of Cr(VI) by CS/Zeo film occurred by chelation and electrostatic interaction with the amine groups of CS and the reactive forms of Cr(VI) [14,18]. The diminished adsorption of Cr(VI) by CS/Zeo film at pH 6.0 than pH 4.0 is suggest to be due to a reduction of protonated reactive forms in CS. The release of Cr(VI) to the solution between 4–8 h at pH 4.0 by CS/Zeo film is suggested by the solubility of a part of the film during the assay (photographic data not shown), however the contact time up to 12 h favored the chelation of the majority of Cr(VI) from the solution when compared with pH 6.0 under the same Cr(VI) concentration conditions. During the desorption experiments there was no desorption of Cr(VI) in distilled water, which confirmed the strong interaction of CS/Zeo film and the Cr(VI).

### 2.3. Adsorption isotherms

Equilibrium isotherms are used to upscale laboratorial batch experiments to a pilot or industrial scale. The equilibrium data of metallic ions adsorption by CS/Zeo film was modeled to the following linearized forms: Ce/Qe = 1/amb + (1/am) Ce (Langmuir linear equation); logQe = log Kf + (1/n) logCe (Freundlich linear equation); ln (KR(Ce/Qe) – 1) = aR ln (Ce) + ln (β) (Redlich-Peterson linear equation) (Table 2).

**Table 2:** Adsorption constants and fitting regression parameters for the isotherm models studied for Cr(VI) onto chitosan/zeolite film, at pH 4.0 and 6.0.

Variables	Equilibrium Isotherms Models
Q_max_ = 66.8000, b = 0.0030, R^2^ = 0.9940 (pH 4.0)	Langmuir
Q_max_ = 21.0370, b = 0.0081, R^2^ = 0.9830 (pH 6.0)	
K_f_ = 0.3679, n = 1.2548, R^2^ = 0.9893 (pH4.0)	Freudlich
k_f_ = 0.6949, n = 1.8027, R^2^ = 0.9977 (pH 6.0)	
K_r_ = 0.2004, a_R_ = 0.0030, β = 1.0000, R^2^ = 0.9940 (pH 4.0)	Redlich-Peterson

**Figure 5:**
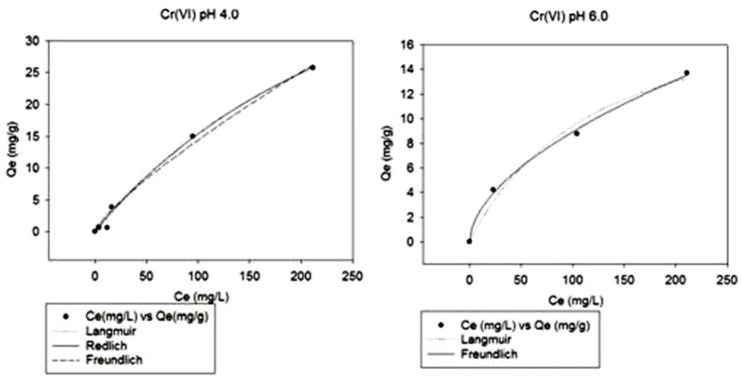
Comparison between experimental data of Cr(VI) adsorption on chitosan/zeolite film and those predicted by the three models tested, at two pH sets.

All models tested showed a good R parameter (~0.98–0.99) depending on initial pH. At initial pH 4.0 both Langmuir and Redlich-Peterson models showed the best fit; at pH 6.0, the Freundlich model showed a better fit. The assay at pH 6.0 did not allow the fitting of the Redlich-Peterson model as shown in Figure 5. These results indicate that adsorption at pH 4.0 occurred, preferably, by means of formation of a monolayer on the film surface. This linkage at pH 4.0 is supposed to happen by electrostatic interaction and occurred for a high concentration of Cr(VI) in solution. At pH 6.0 and in a low concentration range of Cr(VI), it is suggested that the adsorption occurs by chelation on energetically heterogeneous active sites present on the film surface, as shown in Figure 5 [5,18,19,24].

## 3. Experimental

### 3.1. Materials

Chitosan (degree of deacetylation (DD) ~90%) was purchased from Aqua Premier Company, Thailand. Zeolite 13X (hydrophilic; particle diameter = 5–8 mm; pore diameter = 13 Å) was purchased from Xiamen Zhongzhao Imp. & Exp. The zeolite was calcined at 500 °C during 8 h under a dry air stream prior to use. Acetic acid (99%) was purchased from Sigma-Aldirich (St Louis, MO, USA) and diluted to 1% (v/v) in distilled water. The standard metal solution was prepared starting with 2.828 g/L of K_2_Cr_2_O_7_ (Panreac) and pH was adjusted to 4.0 and 6.0 (0.1 mol/L H_2_SO_4_; 0.1 mol/L NaOH).

### 3.2. Film Preparation and Characterization

Solutions of crustacean CS were prepared in acetic acid (1% v/v) and ranged from 0.5–2.5 wt%. CS and CS/Zeo (1:10 w/w) films were prepared as per Liu *et al.* [10]. The percentage of zeolite in chitosan film was selected based on optimal performance in the pervaporation and direct methanol fuel cell (DMFC) process [10,11]. All films were kept in an dessicator for a minimum of 24 h before use.

The films were characterized in terms of water solubility and in terms of structure by X-ray diffractometry, thermogravimetric analysis (TGA - Shimadzu, model TGA-50)) and by Fourier Transmittance Infra-Red spectroscopy (FTIR) obtained from powdered samples on KBr pellets, using a Bomem MB104 spectrometer in the range (4,000–500) cm^−1^ by averaging 20 scans at a maximum resolution of 10 cm^−1^ [12,13,16].

### 3.3. Adsorption/Desorption experiments

The adsorption assays were performed with the addition of only one film per batch (± 50 mg CS; ±298 mg CS/Zeo per film). Control assay was performed with distillated water and pellets of zeolite 1 wt%. The glass bottles with 500 mL of metal solution in different concentration (5–260 mg/L) and with the adsorbent were kept at 25 °C, 150 rpm per 12 h and small aliquots were withdrawn. Total concentrations of the metal were measured by Atomic Absortion (AAS), using a Varian Spectra AA-400. After analysis, the solutions were treated according to Standard Methods [25]. The adsorption uptake was calculated by mass balance. The equilibrium isotherms were fitted to the Langmuir, Freundlich and Redlich-Peterson models. During the batch experiments, the pH of the solutions was not controlled, because the literature reported that the addition of HCl or NaOH to control pH would change the adsorption conditions [26]. Desorption studies were performed *per* Baroni *et al*. [18], with some modifications. The chitosan film was immersed in 100 mL of distilled water for 24 h with stirring at 200 rpm. The metal concentration in solution was measured as previously described.

## 4. Conclusions

This study establishes the ability of CS/Zeo film to adsorb low concentrations of hexavalent chromium from aqueous solutions. The low cost of the matrix manufacture, the good uptake of Cr(VI) at acid pH (17.28 mg/g) and the non desorption of Cr(VI) from the film in water suggests that this combination of chitosan and zeolite could be tested in industrial environments. The industrial approach of this film is suggested to meet the requirements of the current environmental legislation.
